# Correction to “Reduced Susceptibility to Phytophthora in Non‐Transgenic Cacao Progeny Through CRISPR–Cas9 Mediated TcNPR3 Mutagenesis”

**DOI:** 10.1111/pbi.70722

**Published:** 2026-07-13

Guiltinan, M.J., Landherr, L., Maximova, S.N., DelVecchio, D., Sebastian, A. and Albert, I. (2026) Reduced Susceptibility to Phytophthora in Non‐Transgenic Cacao Progeny Through CRISPR–Cas9 Mediated TcNPR3 Mutagenesis. Plant Biotechnology Journal 24: 442–454. https://doi.org/10.1111/pbi.70365

In the originally published article, the line designated GM‐249 was identified as the cacao genotype Scavina‐6 (Sca‐6) and was used as a non‐edited reference in the transcriptome analysis and as the pollen parent in one of the reported crosses. Whole‐genome sequencing of the experimental genotypes has since established that GM‐249 is the cacao clone ICS‐1, not Scavina‐6. The misidentification originated as a labeling error on the source plant and was detected only once genome‐scale data became available; the corrected identity has been confirmed by whole‐genome sequence comparison.

The correction changes the genotype label of GM‐249 from “Scavina‐6 (Sca‐6)” to “ICS‐1” in Table 1, the relevant figures and the supplementary materials, and in the description of the cross in which GM‐249 (ICS‐1) served as the pollen parent. A corrected Table 1 accompanies this correction.

**TABLE 1:** (corrected). Genotyping of T. cacao parent lines and seedlings from the crosses Ker‐1L × PSU‐52 and ICS‐1 × PSU‐67. Details from visual, PCR, and sequence analysis and the resulting seedlings. The region between the two sgRNA guide sites was analyzed for base‐pair changes or deletions. Detailed analysis was performed on the core experimental plants (^†^). A positive gel result is noted “+”, absence of a band “−”. Samples with a band for transgenic DNA were not cloned and sequenced (ND, not determined). All seedlings are heterozygous.

Genotype	Plant #	Pod #	Amp. Set 1	Amp. Set 2	Amp. Set 3	Amp. Set 4	EGFP	Allele structure
Parental lines								
^†^PSU Scavina‐6	PSU‐156	2006 bp	−	−	−	−		NPR3
^†^T0 CRTcNPR3 L1	PSU‐52	948 bp	+	+	+	+		1058 bp deletion/10 KB inverted repeat
^†^T0 CRTcNPR3 L2	PSU‐67	2008 bp	+	+	+	+		2 × 1 bp insertions (homozygous)
^†^T0 CR Vector Ctrl	PSU‐173	2006 bp	+	+	+	+		NPR3
^†^Ker1‐L	PSU‐302	2006 bp	−	−	−	−		NPR3
^†^ICS‐1^1^	PSU‐249	2006 bp	−	−	−	−		NPR3
Seedlings from CR‐NPR3 PSU‐52 × Ker1‐L								
^†^F1 Seedling	PSU‐494	1	948 bp	−	−	−	−	1058 bp deletion
^†^F1 Seedling	PSU‐495	2	2008 bp	−	−	−	−	2 × 1 bp insertions
F1 Seedling	PSU‐496	1	2006 bp	−	−	−	−	NPR3
^†^F1 Seedling	PSU‐497	1	964 bp	−	−	−	−	1042 bp deletion
^†^F1 Seedling	PSU‐498	2	948 bp	−	−	−	−	1058 bp deletion
^†^F1 Seedling	PSU‐499	2	964 bp	−	−	−	−	1042 bp deletion
F1 Seedling	PSU‐500	2	~1 kb	+	+	+	+	ND
F1 Seedling	PSU‐501	1	2006 bp	−	−	−	−	NPR3
Seedlings from CR‐NPR3 PSU‐67 × ICS‐1^1^								
F1 Seedling	PSU‐502	2	~1 kb	+	+	+	+	ND
^†^F1 Seedling	PSU‐503	1	1966 bp	−	−	−	−	41 bp deletion; 1 bp insertion
F1 Seedling	PSU‐504	1	2006 bp	−	−	−	−	NPR3
F1 Seedling	PSU‐505	2	2006 bp	−	−	−	−	NPR3
F1 Seedling	PSU‐506	2	~1 kb	+	+	+	+	ND
F1 Seedling	PSU‐507	2	2006 bp	−	+	+	+	ND
^†^F1 Seedling	PSU‐508	2	948 bp	−	−	−	−	1058 bp deletion
F1 Seedling	PSU‐509	2	~1 bp	−	+	+	+	ND
F1 Seedling	PSU‐510	2	~1 kb	+	+	+	+	ND
F1 Seedling	PSU‐511	1	2006 bp	−	−	−	−	NPR3
F1 Seedling	PSU‐512	1	2006 bp	−	−	−	−	NPR3
F1 Seedling	PSU‐513	2	~1 kb	+	+	+	+	ND
F1 Seedling	PSU‐514	2	~1 kb	+	+	+	+	ND
F1 Seedling	PSU‐515	1	2006 bp	−	−	−	−	NPR3

^1^ Corrected from the originally published “Sca‐6 (Scavina‐6).” The line PSU‐249 (GM‐249) was reassigned to ICS‐1 following whole‐genome sequencing; the original designation resulted from a source‐plant labeling error. See accompanying erratum.

^†^ Core experimental plants on which detailed sequence analysis was performed. “+/−” denote presence/absence of a PCR band. ND, not determined (transgene‐positive samples not cloned/sequenced).

This reassignment does not alter the study's principal conclusions. The most strongly supported and reproducible differential‐expression signal derives from comparisons among lines in the PSU‐Scavina‐6 background, which do not involve GM‐249 and are therefore unaffected. The corrected identity indicates that comparisons involving GM‐249 span a more genetically divergent background than originally stated, which is consistent with, and reinforces, the paper's caution that genetic‐background heterogeneity contributes substantially to the observed transcriptional variation. No quantitative result, figure, or conclusion other than the GM‐249 genotype label requires revision.

We apologize for this error.

